# From Epigenetic Dysregulation to Therapeutic Reprogramming in Endometrial Cancer: A State–Response Framework for Treatment Resistance

**DOI:** 10.3390/ijms27104371

**Published:** 2026-05-14

**Authors:** Manyata Srivastava, Shishir Singh, Pratik Kumar, Neha Moond, Ankita Chouhan, Komal Mann, Sarita Kumari, Monisha Banerjee, Atar Singh Kushwah

**Affiliations:** 1 Translational Oncology & Women’s Health, Department of Biosciences, Manipal University Jaipur, Jaipur 303007, India; manyata.2501049205@muj.manipal.edu (M.S.);; 2 Division of Immunobiology, Cincinnati Children’s Hospital Medical Center, Cincinnati, OH 45229, USA; shishir.singh@cchmc.org; 3 Gynae Surgical Oncology, Max Institute of Cancer Care, Max Healthcare, Delhi 110075, India; sarita2325@gmail.com; 4 Molecular & Human Genetics Laboratory, Department of Zoology, University of Lucknow, Lucknow 226007, India; monishabanerjee30@gmail.com

**Keywords:** endometrial cancer, epigenetic reprogramming, therapeutic resistance, treatment response, DNA methylation, HDAC inhibitors, immunotherapy sensitization

## Abstract

Endometrial cancer (EC) is increasingly recognized as a heterogeneous disease, yet current treatment strategies often fail to explain why tumors with similar molecular profiles respond differently or develop resistance. This gap points to regulatory mechanisms beyond static genomic alterations. Epigenetic dysregulation through DNA methylation, histone modification, and non-coding RNA (ncRNAs) networks acts as a dynamic and reversible system that governs how tumors adapt under therapeutic pressure. In EC, alterations affecting key regulators such as *MLH1*, *PTEN*, and hormone receptors directly influence sensitivity to immunotherapy, targeted therapy, and endocrine treatment, defining treatment-responsive and treatment-resistant states. These observations shift the role of epigenetics from a descriptive feature of tumor biology to a determinant of therapeutic behaviour. Epigenetic states influence immune recognition, pathway activation, and cell cycle control, thereby shaping response to chemotherapy and immune checkpoint blockade. Biomarkers derived from these alterations, including methylation signatures and circulating RNAs, offer opportunities for patient stratification and longitudinal monitoring of treatment response. Therapeutically, targeting epigenetic regulators provides a strategy to reverse resistance and restore treatment sensitivity. DNA methyltransferase and histone deacetylase inhibitors, particularly in combination with established therapies, have shown potential to enhance treatment efficacy. Emerging approaches, including locus-specific epigenetic editing and liquid biopsy–guided monitoring, further support adaptive treatment strategies. Integrating epigenetic reprogramming into clinical decision-making offers a practical path toward improving treatment response and overcoming resistance in EC. Here, we propose an Epigenetic State–Response Framework (ESRF) in which dynamic epigenetic states define treatment-sensitive and resistant phenotypes, map to specific therapeutic vulnerabilities, and can be actively reprogrammed to restore treatment response.

## 1. Introduction

Endometrial cancer (EC) is increasingly recognized as a biologically heterogeneous disease; however, clinical management continues to face a fundamental limitation in explaining why tumors with comparable molecular classifications exhibit markedly different responses to therapy [1,2]. Although genomic profiling has improved disease stratification, it does not fully capture the dynamic nature of treatment response, particularly the emergence of acquired resistance following initially effective therapy [3]. This gap reflects an incomplete understanding of regulatory mechanisms that govern tumor adaptation [4] under therapeutic pressure.

EC remains one of the most common gynecological malignancies worldwide, with increasing incidence across diverse populations. While early-stage disease is often associated with favorable prognosis, advanced and recurrent EC is characterized by limited therapeutic options and poor clinical outcomes [5]. Molecular classification has improved risk stratification; however, substantial variability in treatment response persists within defined subgroups, underscoring the need to understand regulatory layers beyond genomic alterations [3,6].

Epigenetic regulation provides a mechanistic framework to address this limitation. In contrast to fixed genetic alterations, epigenetic states are dynamic, reversible, and responsive to microenvironmental and therapeutic cues, enabling transcriptional reprogramming without changes in DNA sequence [7,8]. Epigenetic mechanisms including DNA methylation, post-translational histone modifications, ATP-dependent chromatin remodeling, and non-coding RNA (ncRNAs)–mediated regulation, play central roles in orchestrating gene expression programs in cancer [9,10]. In EC, these processes contribute to tumorigenesis through silencing of tumor suppressor genes (e.g., *MLH1*, *PTEN*), activation of oncogenic pathways such as PI3K/AKT signaling, and modulation of cellular differentiation and proliferation states [3,11,12,13]. Importantly, the reversible nature of these modifications distinguishes them from genetic lesions and provides a basis for therapeutic targeting [14,15,16].

Beyond their role in tumor initiation and progression, epigenetic alterations function as active regulators of therapeutic response [17]. Aberrant DNA methylation patterns, histone modification landscapes, and ncRNAs networks influence key processes including cell cycle control, apoptotic signaling, epithelial–mesenchymal transition, and immune evasion [18,19,20]. For instance, epigenetic silencing of mismatch repair genes such as *MLH1* contributes to microsatellite instability and altered neoantigen landscapes, thereby modulating sensitivity to immune checkpoint inhibitors [3,21]. Similarly, epigenetic regulation of estrogen and progesterone receptor expression directly impacts endocrine responsiveness, while chromatin state alterations influence accessibility of transcriptional programs governing therapeutic resistance [22,23].

These observations redefine epigenetics as an active regulator of therapeutic behavior rather than a passive layer of tumor biology [24]. Epigenetic biomarkers, including DNA methylation signatures and circulating ncRNAs, are emerging as clinically relevant tools for predicting treatment response and monitoring tumor evolution in real time [25,26]. Concurrently, pharmacological targeting of epigenetic regulators, such as DNA methyltransferases and histone deacetylases, offers a strategy to reverse transcriptional repression, restore pathway function, and re-sensitize resistant tumors to therapy [27,28].

Beyond tumor-intrinsic mechanisms, variability in treatment response is influenced by limitations in existing clinical classification frameworks. Current classification systems in EC do not fully capture disease heterogeneity or predict therapeutic outcomes, highlighting the need for integrative models that incorporate dynamic molecular regulation [1,2]. These layers highlight that therapeutic response should be interpreted as an emergent property arising from the interaction between dynamic tumor states and clinical decision environments. This integrated framework connects epigenetically defined tumor states with their functional consequences and corresponding therapeutic reprogramming strategies.

In this context, epigenetic reprogramming represents a mechanistically grounded and clinically actionable approach to overcoming therapeutic resistance in EC [15,29]. This review integrates current understanding of epigenetic regulation with therapeutic response dynamics, highlighting functionally actionable targets and emerging strategies to incorporate epigenetic interventions into precision oncology frameworks. Importantly, we propose an Epigenetic State–Response Framework (ESRF) that organizes EC into dynamic epigenetic states linked to (i) functional tumor behavior, (ii) therapeutic response phenotypes, and (iii) reprogrammable intervention strategies. This framework provides a mechanistic bridge between molecular regulation and clinically actionable treatment adaptation.

Several recent studies and reviews have highlighted the roles of chromatin regulation, epigenetic dysregulation, and molecular heterogeneity in EC progression and therapeutic response [2,30]. However, these studies primarily describe epigenetic alterations as molecular characteristics of tumor biology rather than as dynamic determinants of therapeutic behavior. In contrast, the present review introduces the ESRF, which conceptualizes EC as a system of reversible epigenetic states linked to treatment sensitivity, resistance evolution, immune responsiveness, and re-sensitization potential. By integrating mechanistic epigenetic regulation with adaptive therapeutic response, ESRF provides a translational framework for state-guided precision oncology in EC.

Unlike existing descriptive models of epigenetic dysregulation, the ESRF framework introduces a state-based, functionally mapped structure that links regulatory mechanisms to therapeutic response and reversibility. This enables not only the classification of tumor behavior but also the prediction of re-sensitization potential under targeted epigenetic intervention.

## 2. Epigenetic Mechanisms as Determinants of Therapeutic Response in Endometrial Cancer

Epigenetic processes play a central role in regulating gene expression programs that govern tumor behavior in EC [18,30]. Beyond their contribution to tumor initiation and progression, these mechanisms function as dynamic regulators of how cancer cells respond to therapeutic pressure [31,32]. DNA methylation, histone modifications, ncRNAs networks, and chromatin remodeling collectively shape transcriptional states that define treatment-sensitive and treatment-resistant phenotypes [9,24].

DNA methylation is one of the most extensively characterized epigenetic mechanisms in EC [18,30,33]. It involves the covalent addition of a methyl group to cytosine residues within CpG islands, typically resulting in transcriptional repression [9,34]. Aberrant promoter hypermethylation of tumor suppressor genes such as *MLH1* leads to gene silencing, contributing to mismatch repair deficiency, microsatellite instability (MSI), and genomic instability [21,35,36]. In the therapeutic context, these alterations have direct clinical relevance because of mismatch repair (MMR) deficiency in MSI-high tumors, which leads to the progressive accumulation of insertion or deletion mutations, specifically within repetitive microsatellite regions of the genome [21,37]. These mutations often generate aberrant proteins and frameshift mutations that the immune system recognizes as neoantigens. When tumor neoantigen burden increases, it enhances tumor immunogenicity and promotes infiltration of cytotoxic CD8^+^ T-cells and other immune effector populations into the tumor microenvironment [38,39]. Hence, MSI-high tumors frequently exhibit an inflamed immune phenotype characterized by increased expression of immune checkpoint molecules, such as CTLA-4, PD-1, and PD-L1, which arise as adaptive immune evasion mechanisms. MSI-high tumors are especially vulnerable to immune checkpoint blockade therapy, including anti-PD-1 and anti-PD-L1 inhibitors [40,41]. In EC, MSI-high or MMR-deficient tumors have revealed improved clinical responses to immunotherapeutic drugs like pembrolizumab and dostarlimab, highlighting the translational significance of immune regulation associated with epigenetic and genome instability [42].

Conversely, global hypomethylation can activate oncogenes and promote chromosomal instability, further contributing to tumor heterogeneity and adaptive resistance [7,8]. Across these mechanisms, epigenetic regulation converges on a limited set of functional outputs—DNA repair capacity, apoptotic threshold, immune visibility, and metabolic adaptation, which collectively define treatment response states within the ESRF model.

Histone modifications represent another critical layer of epigenetic regulation, influencing chromatin architecture and transcriptional accessibility [42,43,44,45,46]. Post-translational modifications such as acetylation, methylation, phosphorylation, and ubiquitination regulate the balance between transcriptionally active and repressed chromatin states [20,45,46,47]. Histone acetylation mediated by histone acetyltransferases (HATs) promotes transcriptional activation, whereas histone deacetylation by histone deacetylases (HDACs) leads to chromatin condensation and gene silencing [28,48]. In EC, dysregulated histone modification patterns contribute to altered expression of genes involved in cell cycle regulation, apoptosis, and differentiation [12,18,49]. From a therapeutic perspective, these chromatin states influence accessibility of drug-responsive pathways and have been implicated in resistance to both cytotoxic and targeted therapies [50]. Recent mechanistic studies in EC have further demonstrated that inhibition of PRMT6 activates interferon signaling and promotes apoptosis through histone modification–mediated transcriptional reprogramming, highlighting the direct role of chromatin regulators in shaping therapeutic vulnerability [20]. Consistently, emerging evidence indicates that targeting PRMT family–associated epigenetic signaling may additionally enhance anti-tumor immunity through activation of innate immune pathways, including cGAS/STING signaling [51].

Non-coding RNAs (ncRNAs), including microRNAs (miRNAs) and long non-coding RNAs (lncRNAs), provide an additional regulatory layer that modulates gene expression at transcriptional and post-transcriptional levels [8,9,52,53]. miRNAs regulate mRNA stability and translation by binding to the 3′ untranslated region of target transcripts [19,36,54] while lncRNAs influence gene expression through chromatin interactions, transcriptional interference, and protein scaffolding [55,56]. In EC, dysregulation of these ncRNAs contributes to tumor proliferation, invasion, epithelial–mesenchymal transition (EMT), and metastatic progression [16,36,53,57,58,59]. Similarly, methylation-mediated silencing of tumor suppressor miRNAs such as miR-124, miR-137, and miR-152 has been shown to promote EMT, invasion, and metastatic progression in EC through dysregulation of oncogenic signaling pathways [19,54,57]. Importantly, ncRNA-mediated networks also influence therapeutic response by regulating apoptosis pathways, drug metabolism, and immune signaling, thereby contributing to resistance phenotypes [29].

Chromatin remodeling further modulates gene accessibility through ATP-dependent nucleosome repositioning [17,46]. Remodeling complexes such as SWI/SNF regulate transcription factor binding and maintain chromatin organization. Dysregulation or mutation of these complexes in EC alters transcriptional programs that control tumor growth and differentiation [22]. Emerging evidence further indicates that *ARID1A*-deficient EC exhibits selective vulnerability to *PRMT5*-targeted approaches, supporting the concept that chromatin remodeling defects may generate therapeutically actionable epigenetic dependencies [60]. These changes not only contribute to tumor progression but also affect the accessibility of genomic regions involved in drug response, thereby influencing therapeutic efficacy.

Collectively, these epigenetic mechanisms do not operate in isolation but form an integrated regulatory network that defines tumor plasticity [61]. Epigenetic alterations enable reversible transitions between distinct transcriptional states, allowing tumor cells to adapt to environmental and therapeutic stress [62]. This plasticity underlies the emergence of treatment-resistant subpopulations and represents a fundamental barrier to durable therapeutic responses [63].

Importantly, the reversible nature of epigenetic modifications distinguishes them from genetic alterations and provides a mechanistic basis for therapeutic intervention [14,15]. Epigenetic drugs, including DNA methyltransferase and histone deacetylase inhibitors, have demonstrated the ability to reprogram resistant tumor states, restore expression of silenced tumor suppressor genes, and enhance sensitivity to chemotherapy and immunotherapy [27,64]. In addition, combination strategies integrating epigenetic therapy with conventional treatments or immune checkpoint inhibitors have shown promise in overcoming resistance and improving clinical outcomes [65,66].

Thus, epigenetic dysregulation in EC represents not only a driver of tumorigenesis but also a dynamic and targetable determinant of therapeutic response. Understanding how these mechanisms regulate treatment sensitivity and resistance provides a foundation for developing strategies aimed at epigenetic reprogramming and adaptive cancer therapy.

## 3. Therapeutically Actionable Epigenetic Targets in Endometrial Cancer

Epigenetic regulation of key molecular pathways in EC provides a direct mechanistic link between gene expression control and therapeutic response [18,30]. Specific epigenetically altered targets function as nodal regulators of DNA repair, oncogenic signaling, cell cycle progression, and hormone responsiveness, thereby defining clinically actionable tumor states [61,63,67]. These targets, along with their mechanistic roles and therapeutic implications, are summarized in Table 1.

Epigenetic silencing of *MLH1* represents a prototypical example of how DNA methylation directly shapes therapeutic response. Promoter hypermethylation of *MLH1* leads to transcriptional repression, resulting in impaired DNA mismatch repair (MMR) activity and the development of microsatellite instability (MSI) [3,40,68]. Functionally, this defect increases mutational burden and tumor heterogeneity [40]. However, from a therapeutic perspective, *MSI*-high tumors exhibit enhanced neoantigen presentation and increased immunogenicity, rendering them more responsive to immune checkpoint inhibitors [37,40]. Thus, *MLH1* methylation status serves not only as a marker of genomic instability but also as a determinant of immunotherapy sensitivity, linking epigenetic regulation to immune-based treatment strategies [37,69] (Table 1).

The tumor suppressor *PTEN* is another critical epigenetically regulated target with direct implications for therapeutic resistance. Epigenetic silencing of *PTEN* through promoter methylation or chromatin-mediated repression leads to constitutive activation of the PI3K/AKT signaling pathway [11,12]. This activation promotes cell survival, proliferation, and metabolic reprogramming, while simultaneously reducing apoptotic responsiveness [11]. Functionally, downstream activation of mTOR and associated signaling cascades supports tumor progression and contributes to resistance against targeted and cytotoxic therapies [70]. In this context, *PTEN* loss defines a therapeutically relevant signaling axis that integrates epigenetic repression with oncogenic pathway activation, providing a rationale for PI3K/AKT/mTOR-targeted interventions in combination with epigenetic modulators to restore pathway control [15,71] (Table 1).

Epigenetic inactivation of *CDKN2A* (p16) disrupts cell cycle regulation and represents a key mechanism underlying uncontrolled proliferation in EC. Promoter hypermethylation leads to loss of p16 expression, impairing its ability to inhibit cyclin-dependent kinases CDK4/6 and facilitating unchecked progression from the G1 to S phase [72,73]. This deregulation not only enhances proliferative capacity but also compromises cell cycle checkpoints, allowing replication of damaged DNA and contributing to genomic instability [72]. From a therapeutic standpoint, loss of p16 function defines a cell cycle-dysregulated tumor state that may influence sensitivity to CDK4/6 inhibitors and other anti-proliferative strategies, highlighting the role of epigenetic control in shaping treatment response [73] (Table 1).

Hormonal signaling pathways, mediated by estrogen receptor (ER) and progesterone receptor (PR), are tightly regulated by epigenetic mechanisms and play a central role in endometrial tumor biology [5,74]. DNA methylation and histone modifications alter ER and PR expression, thereby modulating hormone-responsive transcriptional programs [22,23,74]. Reduced PR expression, often associated with promoter methylation, impairs progesterone-mediated anti-proliferative signaling and permits estrogen-driven tumor growth [23]. Similarly, alterations in ER-dependent transcriptional programs influence cellular differentiation and proliferation [22]. These epigenetic changes define a hormone-resistant tumor state with direct clinical implications, as they determine responsiveness to endocrine therapies. Restoration of ER/PR expression through epigenetic modulation may re-sensitize tumors to hormonal treatment, providing a rationale for integrating epigenetic therapies with endocrine strategies [22,75,76] (Table 1). Within the ESRF model, these targets do not act independently but define discrete epigenetic states characterized by specific response phenotypes, thereby enabling functional classification of tumors beyond genomic subtypes.

Collectively, these epigenetically regulated targets converge on key signaling pathways governing DNA repair, cell cycle control, oncogenic activation, and hormone responsiveness, thereby defining functional tumor states that determine therapeutic sensitivity [17,77]. As summarized in Table 1, these alterations do not operate in isolation but integrate to shape dynamic transcriptional programs that underlie response to immunotherapy, targeted therapy, and endocrine treatment. This target-level framework provides the mechanistic foundation for epigenetic reprogramming strategies aimed at restoring treatment sensitivity and overcoming resistance, as discussed in the following section. Within the ESRF model, Table 1 defines core epigenetic states through target-level alterations that directly determine therapeutic response phenotypes.

**Table 1:** Therapeutically actionable epigenetic targets defining tumor behavior in endometrial cancer.

Gene/Axis	Epigenetic Alteration	Mechanistic Impact	Functional Outcome in EC	Clinical/Therapeutic Relevance
*MLH1*	Promoter hypermethylation	Transcriptional silencing of MMR pathway	MSI phenotype, increased mutation burden	Predicts response to immune checkpoint inhibitors [3,21,27,51,78]
*PTEN*	Promoter methylation/DNMT-mediated repression	Loss of tumor suppressor and PI3K/AKT activation	Enhanced proliferation, survival, metabolic shift	Targetable via PI3K/mTOR inhibitors; resistance driver [11,12,67]
*CDKN2A* (*p16*)	Promoter hypermethylation	Loss of CDK4/6 inhibition	G1/S checkpoint failure, genomic instability	Predicts aggressive phenotype; CDK4/6 targeting context [67,72]
*ER* (*ESR1*)	DNA methylation/chromatin repression	Altered estrogen signaling transcriptional programs	Dysregulated proliferation and differentiation	Influences endocrine therapy response [22,79]
*PR* (*PGR*)	Epigenetic silencing (MeCP2, methylation)	Loss of progesterone-mediated transcription	Loss of anti-proliferative control	Hormone therapy resistance; re-sensitization potential [23]
miR-124/miR-152/miR-137/miR-192	Hypermethylation-mediated silencing	Post-transcriptional repression of oncogenic targets	EMT, invasion, proliferation	Biomarkers + therapeutic restoration targets [19,36,52,54,57]
HDAC axis	Overexpression/aberrant activity	Chromatin compaction, transcriptional repression	Reduced apoptosis, therapy resistance	Targetable via HDAC inhibitors [28,48]
*ARID1A*/*SWI-SNF*	Chromatin remodeling disruption	Loss of nucleosome regulation	Transcriptional dysregulation, lineage plasticity	Synthetic lethality (PRMT5, EZH2 contexts) [60,80]

## 4. Epigenetic Biomarkers for Treatment Stratification and Therapeutic Monitoring

Epigenetic biomarkers are increasingly recognized not only as tools for cancer detection but as clinically relevant determinants of therapeutic response and disease monitoring in EC [35,81,82]. Given the dynamic and reversible nature of epigenetic alterations, these biomarkers provide a functional readout of tumor state, enabling real-time assessment of treatment sensitivity and resistance [7,82].

DNA methylation signatures represent one of the most robust classes of epigenetic biomarkers in EC [30,81]. Aberrant methylation patterns in tumor suppressor genes, including *MLH1*, can be detected in tumor tissue as well as in circulating cell-free DNA (cfDNA) [3,25]. While these alterations serve as early indicators of malignant transformation [81,83], their clinical relevance extends beyond diagnosis. For example, *MLH1* methylation status reflects mismatch repair deficiency and microsatellite instability, which are directly associated with responsiveness to immune checkpoint inhibitors. Thus, methylation profiling provides a mechanistic link between tumor biology and immunotherapy selection.

Circulating ncRNAs, particularly miRNAs, offer a minimally invasive approach to monitoring tumor dynamics [26,84]. Distinct miRNA expression profiles have demonstrated high sensitivity and specificity in detecting EC [54,57]. More importantly, these circulating RNAs exhibit dynamic changes during treatment, reflecting alterations in tumor burden, pathway activation, and therapeutic response [19,85,86]. As such, they have potential utility as longitudinal biomarkers for assessing treatment efficacy and detecting early emergence of resistance [26].

Prognostic epigenetic biomarkers further refine clinical risk stratification by linking molecular alterations to disease progression and survival [2,5,49,87]. Hypermethylation of tumor suppressor genes is frequently associated with aggressive tumor phenotypes [45,88], including higher grade, deeper myometrial invasion, and increased metastatic potential [12,59,61,67]. Similarly, dysregulated expression of miRNAs and lncRNAs has been correlated with recurrence risk and reduced overall survival [55,56]. While these features inform prognosis, their integration with therapeutic decision-making is critical for guiding treatment intensity and follow-up strategies [2].

Predictive epigenetic biomarkers are particularly relevant for precision oncology, as they inform treatment selection based on functional tumor states [15]. Methylation patterns affecting DNA repair pathways, such as MLH1, can predict response to immunotherapy [37], while epigenetic regulation of oncogenic signaling pathways influences sensitivity to targeted agents and chemotherapy [11,12]. In addition, dynamic changes in circulating miRNAs during therapy provide insight into treatment response and evolving resistance mechanisms, offering an advantage over static genomic markers [26,85].

Importantly, the clinical utility of epigenetic biomarkers lies in their ability to capture tumor plasticity [17,77]. Unlike genetic alterations, which represent fixed events, epigenetic changes reflect the ongoing adaptation of tumor cells under therapeutic pressure [7]. This makes them particularly valuable for longitudinal monitoring using minimally invasive approaches such as liquid biopsy, enabling repeated assessment of tumor evolution without the need for serial tissue sampling [25,26].

Integration of epigenetic biomarkers into clinical workflows has the potential to transform treatment strategies in EC [2]. By linking molecular alterations to therapeutic response, these biomarkers support patient stratification, guide selection of targeted and immunotherapies, and enable real-time monitoring of treatment efficacy [15,76]. As such, they form a critical component of epigenetic reprogramming strategies aimed at overcoming resistance and improving clinical outcomes [15].

## 5. Epigenetic Reprogramming Strategies: Reversing Resistance and Enhancing Therapeutic Response

Within the ESRF model therapeutic resistance in EC emerges from transitions into epigenetically defined drug-tolerant states rather than fixed genetic alterations [77,89]. Tumor cells can transition into drug-tolerant transcriptional states through reversible epigenetic modifications, allowing survival under therapeutic pressure [62]. This adaptive capacity shifts the therapeutic paradigm from targeting fixed oncogenic drivers to reprogramming dynamic gene expression states that govern treatment sensitivity [7,24]. Building on this framework, therapeutic strategies targeting epigenetic regulators aim to disrupt or reprogram these dynamic tumor states [76], translating mechanistic insights into clinically actionable interventions (Table 2).

DNA methyltransferase (DNMT) inhibitors, such as Azacitidine and Decitabine, represent a key class of agents capable of reversing epigenetic repression [71,90]. By inhibiting DNA methylation, these agents restore expression of silenced tumor suppressor genes involved in DNA damage response, apoptosis, and immune signaling [27,65,69]. In EC models, DNMT inhibition has additionally been associated with restoration of mismatch repair pathway activity and enhanced interferon-mediated immune signaling, thereby increasing susceptibility to immune checkpoint blockade [27,65]. In EC, DNMT inhibition has been shown to enhance tumor immunogenicity through induction of neoantigen expression and activation of interferon signaling pathways, thereby increasing sensitivity to immune checkpoint blockade [20,37,91,92]. This establishes DNMT inhibitors not only as cytotoxic modulators but as agents of immune reprogramming [65,93]. This mechanism of transcriptional reactivation and immune modulation through DNMT inhibition is summarized in Table 2.

Histone deacetylases (HDACs) provide a complementary strategy by altering chromatin accessibility and transcriptional output [48]. Agents such as vorinostat and romidepsin promote histone acetylation, leading to a more open chromatin configuration and reactivation of genes regulating cell cycle arrest, apoptosis, and differentiation [28,94]. In EC, HDAC inhibition has been associated with suppression of tumor growth and enhanced responsiveness to cytotoxic and targeted therapies [11,64]. In addition to histone targets, these agents modulate acetylation of non-histone proteins, thereby influencing signaling pathways involved in cell survival, stress response, and immune interactions [48,64]. These chromatin-mediated effects and their therapeutic implications are outlined in Table 2.

A central concept emerging from these observations is epigenetic priming, in which epigenetic therapies are used to recondition tumor cells prior to or in combination with other treatments [29]. DNMT and HDAC inhibitors can sensitize tumors to chemotherapy by restoring apoptotic competence and reversing drug-tolerant states [64,95]. Similarly, epigenetic modulation of antigen presentation machinery and immune signaling enhances the efficacy of immune checkpoint inhibitors by increasing T-cell infiltration and tumor visibility to the immune system [66]. In EC, such combination strategies have shown particular promise in tumors characterized by microsatellite instability or immune-evasive phenotypes [6,81]. This concept of epigenetic priming, linking chromatin reprogramming to restoration of treatment sensitivity, is a key component of the therapeutic strategies summarized in Table 2. This establishes a temporal dimension to therapy, where epigenetic agents function not only as modifiers of transcription but as state-transition catalysts that shift tumors from resistant to treatment-permissive configurations.

These combination approaches highlight the role of epigenetic therapies as modulators of therapeutic response rather than stand-alone treatments [96]. Importantly, these interventions are directly linked to the epigenetically defined tumor states described in Table 1, reinforcing the connection between target-level dysregulation and therapeutic reprogramming. By targeting multiple regulatory layers simultaneously, epigenetic reprogramming can overcome resistance mechanisms that limit the durability of conventional therapies [4,77]. Importantly, the timing, sequencing, and dosing of epigenetic agents are critical determinants of clinical efficacy, as inappropriate scheduling may fail to achieve effective re-sensitization or may increase toxicity. These combinatorial strategies, integrating epigenetic modulation with chemotherapy and immunotherapy, are systematically represented in Table 2.

Personalized epigenetic therapy represents an emerging direction in precision oncology [97]. Integration of molecular profiling with epigenetic signatures enables the identification of tumors characterized by transcriptional repression rather than irreversible genetic loss [2,3]. In such contexts, restoring gene expression can produce a significant therapeutic benefit [71]. Furthermore, epigenetic biomarkers can guide patient selection and enable real-time monitoring of treatment response, allowing dynamic adaptation of therapeutic strategies [98].

Collectively, these approaches position epigenetic therapies as modulators of dynamic tumor states rather than stand-alone interventions [15]. As summarized in Table 2, targeting epigenetic regulators enables reprogramming of transcriptional networks governing DNA repair, signaling pathways, and immune interactions, thereby restoring treatment sensitivity and overcoming resistance. This therapeutic framework establishes a direct mechanistic continuum from epigenetic dysregulation to clinically actionable intervention strategies [7]. Table 2 operationalizes the ESRF model by mapping epigenetic states to reprogramming strategies capable of restoring therapeutic sensitivity.

**Table 2:** Epigenetic therapeutic strategies for reprogramming tumor states in endometrial cancer.

Strategy	Mechanism	Agents/Tools	Functional Impact	EC-Specific Evidence	Clinical/Translational Relevance
DNMT inhibition	DNA demethylation led to gene reactivation	Azacitidine, Decitabine	Restores *MLH1*, tumor suppressors	Induces apoptosis, MMR restoration	Sensitizes to immunotherapy [27,65,68]
HDAC inhibition	Chromatin relaxation	Vorinostat, Romidepsin	Reactivates apoptosis and cell cycle genes	Growth inhibition in EC models	Enhances chemo/targeted therapy response [28,64]
Epigenetic priming	Pre-treatment chromatin reprogramming	DNMT + HDAC combinations	Reverses drug-tolerant state	Chromatin-mediated reversible resistance states	Improves downstream therapy response [62,77]
Immuno-epigenetic therapy	Viral mimicry + interferon activation	DNMT inhibitors + ICI	Enhances antigen presentation	Induces immune activation in tumors	Converts “cold” to “hot” tumors [65,66]
PI3K pathway targeting	PTEN-axis restoration	PI3K/mTOR inhibitors + epigenetic drugs	Dual pathway suppression	*PTEN*-silenced EC models	Overcomes resistance [11,12]
CRISPR epigenetic editing	Locus-specific methylation/acetylation control	dCas9-based systems	Precise gene activation/silencing	*MLH1* modulation and pathway control	Future precision therapy platform [99,100]
Liquid biopsy–guided therapy	ctDNA methylation + circulating RNA	cfDNA/miRNA profiling	Real-time tumor state monitoring	Validated in EC (ctDNA monitoring)	Enables adaptive therapy [25,98]

## 6. Epigenetic Plasticity, Resistance Dynamics, and Emerging Therapeutic Directions

**Figure 1:**
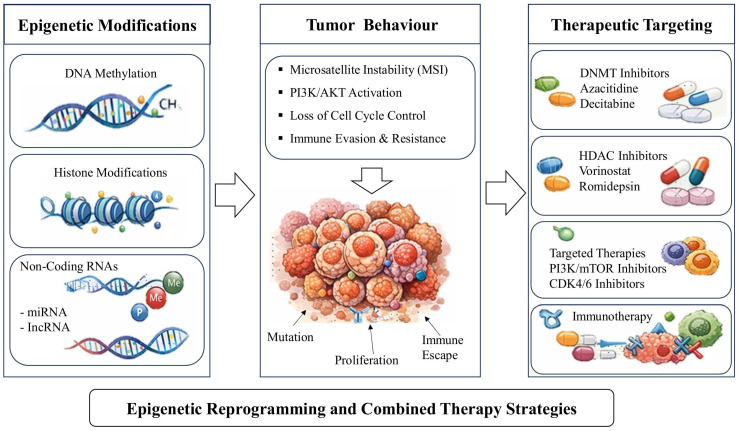
Epigenetic regulation of tumor plasticity and its translation into therapeutic response in endometrial cancer. This figure illustrates a multi-layer framework linking epigenetic regulation to tumor behavior and therapeutic response in EC. Epigenetic alterations—including DNA methylation, histone modifications, chromatin remodeling, and ncRNAs networks—drive dynamic tumor states characterized by genomic instability, oncogenic signaling activation, cell cycle dysregulation, and immune evasion. These states remain functionally plastic, influencing chromatin accessibility, DNA damage sensitivity, and immune activation, thereby shaping responsiveness to therapy. Therapeutic strategies targeting epigenetic regulators, signaling pathways, and immune checkpoints aim to reprogram resistant tumor states toward re-sensitization. Importantly, these regulatory layers and their functional consequences correspond directly to the multi-layer epigenetic architecture summarized in Table 3, which defines the mechanistic basis of tumor plasticity and resistance dynamics. The clinical translation of these dynamic states occurs within a parallel layer defined by limitations in current classification systems and patient-level decision dynamics, which together influence the timing, selection, and effectiveness of therapeutic interventions. This integrated model highlights the need for combining epigenetic biomarkers with adaptive clinical frameworks to achieve durable treatment outcomes.

Within the ESRF model epigenetic plasticity represents the core mechanism enabling transitions between treatment-sensitive and treatment-resistant states [7,17,18]. Under therapeutic pressure, tumor cells can adopt drug-tolerant phenotypes characterized by altered gene expression programs that support survival, metabolic adaptation, and immune evasion [51,53,62,77]. These adaptive transitions allow subpopulations of tumor cells to persist despite initial treatment response, ultimately leading to disease relapse and progression [101]. This systems-level organization of epigenetic regulation is conceptually illustrated in Figure 1 and mechanistically detailed in Table 3.

This dynamic model reframes treatment failure as a consequence of epigenetic state transitions rather than clonal selection alone, where tumor cells reversibly shift between transcriptional configurations that determine therapeutic sensitivity [77,102]. Epigenetically mediated switching between treatment-sensitive and treatment-resistant states contributes to intratumoral heterogeneity and represents a major barrier to durable therapeutic outcomes [17]. Importantly, these transitions are reversible, providing an opportunity to therapeutically reprogram resistant tumor cells back into treatment-responsive states. These reversible transitions are governed by interconnected epigenetic regulatory axes [9] integrating DNA methylation, chromatin remodeling, and ncRNAs networks (Table 3). This coordinated regulatory architecture establishes a mechanistic basis for therapeutic reprogramming, positioning epigenetic plasticity as both the driver of resistance and the entry point for its reversal.

Re-sensitization strategies are therefore increasingly focused on targeting epigenetic regulators to reverse transcriptional repression and restore pathway activity [15,20]. DNA methyltransferase and histone deacetylase inhibitors have demonstrated the ability to reprogram resistant tumor cells by reactivating genes involved in apoptosis, DNA damage response, and immune signaling [11,64,103]. Sequential and combination treatment approaches have shown potential to restore sensitivity to chemotherapy, targeted therapy, and immunotherapy [29]. However, clinical efficacy depends critically on treatment scheduling, dosing, and integration with existing therapeutic regimens, highlighting the need for optimized adaptive treatment strategies [7]. These re-sensitization strategies act by targeting the regulatory layers that define drug-tolerant states, reinforcing the multi-layer epigenetic framework summarized in Table 3.

Emerging technologies are further advancing the precision of epigenetic intervention. CRISPR-based epigenetic editing enables locus-specific modulation of gene expression without altering the underlying DNA sequence [100,104]. Catalytically inactive Cas9 (dCas9) fused with epigenetic modifiers can selectively activate or repress target genes, allowing direct manipulation of disease-relevant transcriptional programs [99]. In EC, this approach provides a platform for dissecting resistance mechanisms and developing targeted strategies to restore tumor suppressor function or suppress oncogenic pathways [105]. Although still in early stages, such technologies represent a shift toward precision epigenetic control rather than global modulation. Such locus-specific interventions provide a direct approach to modulating individual components of the multi-layer epigenetic architecture described in Table 3.

In parallel, liquid biopsy–based approaches are transforming the ability to monitor epigenetic dynamics in real time. Analysis of circulating tumor DNA (ctDNA), DNA methylation patterns, and ncRNAs enables non-invasive assessment of tumor evolution and treatment response [84,106,107]. These approaches allow early detection of resistance-associated epigenetic changes and facilitate timely therapeutic adjustment [25]. Longitudinal monitoring through liquid biopsy provides a practical means to track dynamic tumor states without repeated tissue sampling, supporting adaptive and personalized treatment strategies [108].

Importantly, the clinical manifestation of dynamic tumor states unfolds within a parallel layer of patient-level and system-level constraints. A major limitation in current gynecological disease management is the disconnect between clinical classification systems and underlying biological behavior. Existing staging frameworks, while useful for surgical description, show limited correlation with symptom severity, disease progression, and therapeutic outcomes, reflecting their lack of integration with molecular determinants [6,84]. These approaches enable real-time tracking of dynamic epigenetic states and regulatory pathways that define tumor behavior, as captured in Table 3.

In parallel, translation of these dynamic tumor states into clinical outcomes remains constrained by current classification systems that do not incorporate real-time molecular adaptation [109]. This convergence defines a critical translational interface: even when epigenetic reprogramming strategies successfully alter tumor biology, their clinical effectiveness may depend on timing, accessibility, and integration within real-world decision frameworks. Bridging epigenetic biomarkers with adaptive, patient-centered decision support systems may therefore be essential for achieving truly responsive and durable therapeutic outcomes. Table 3 expands the ESRF model into a multi-layer regulatory architecture, defining how coordinated epigenetic mechanisms sustain tumor plasticity and therapeutic response dynamics.

**Table 3:** Multi-layer epigenetic regulators governing tumor plasticity and therapeutic response in endometrial cancer.

Regulatory Layer	Key EC-Specific Regulators	Mechanistic Role	Functional Outcome	Translational Implication
DNA Methylation	*MLH1*, global CpG dysregulation	Silencing of MMR genes	MSI, genomic instability	Predicts ICI response; DNMT targeting [3,21,27]
Hormone–Epigenetic Axis	ER, PR, MeCP2	Epigenetic control of hormone signaling	Endocrine resistance	Re-sensitization via epigenetic therapy [22,23]
PI3K Epigenetic Control	*PTEN* methylation axis	Loss of tumor suppressor signaling	Survival, metabolic reprogramming	PI3K-targeted combination therapy [11,12]
Chromatin Modification	HDACs, histone acetylation imbalance	Chromatin compaction	Therapy resistance, reduced apoptosis	HDAC inhibitors restore sensitivity [20,28,45,48]
Chromatin Remodeling	*ARID1A*, *SWI*/*SNF*	Nucleosome repositioning defects	Transcriptional instability	Synthetic lethality (axis) [46,60,80]
Non-coding RNA (miRNA)	miR-124, miR-137, miR-152	Post-transcriptional regulation	EMT, invasion, metastasis	Biomarker + therapeutic targets [19,52,54,57]
Non-coding RNA (lncRNA)	NORAD, FAM83H-AS1	Chromatin scaffolding and transcription control	Proliferation, ferroptosis regulation	Emerging therapeutic targets [36,53,55,56]
Epigenetic Plasticity	Drug-tolerant chromatin states	Reversible transcriptional adaptation	Persister cell formation	Targetable via priming strategies [62,77]
Immune–Epigenetic Interface	DNMT–interferon axis	Immune gene silencing	Immune evasion	Enhances ICI response [66,110]
Circulating Epigenetics	ctDNA methylation, circulating miRNA	Real-time tumor state reflection	Monitoring resistance evolution	Adaptive therapy guidance [25,26]

From a translational perspective, the ESRF model provides a clinically adaptable model for integrating dynamic molecular states into therapeutic decision-making in endometrial cancer. Unlike static genomic classifications, ESRF conceptualizes therapeutic response as a reversible and continuously evolving process shaped by epigenetic plasticity under treatment pressure. This framework supports biomarker-guided patient stratification by linking epigenetic alterations, including *MLH1* methylation status, chromatin remodeling defects, and ncRNA signatures, with distinct treatment-responsive and treatment-resistant phenotypes [2,42]. Importantly, integration of ctDNA, methylation-based liquid biopsy approaches, and circulating ncRNAs profiling enables longitudinal monitoring of tumor evolution and early detection of resistance-associated state transitions [25,26,81,106,107,108]. These approaches may facilitate adaptive therapeutic strategies in which treatment timing, sequencing, and combinatorial interventions are modified according to real-time molecular response dynamics rather than baseline classification alone. Within this framework, epigenetic therapies may function not only as direct anticancer agents but also as re-sensitization modulators capable of restoring immune responsiveness, apoptotic signaling, and pathway control prior to chemotherapy, endocrine therapy, or immune checkpoint blockade [29,65,66,90,110]. Collectively, ESRF establishes a translational bridge between epigenetic regulation, dynamic biomarker monitoring, and adaptive precision oncology, thereby supporting the development of state-guided therapeutic strategies aimed at improving the durability of treatment response in endometrial cancer.

Integration of these emerging approaches establishes a framework for adaptive epigenetic oncology, conceptually illustrated in Figure 1 and mechanistically defined in Table 3, in which therapeutic decisions are guided by real-time assessment of tumor state and dynamic response to treatment. As summarized in Table 3, tumor behavior is governed by coordinated interactions across multiple epigenetic regulatory layers linking molecular alterations to functional states and therapeutic outcomes. This systems-level perspective positions epigenetic regulation as a central driver of tumor plasticity and a critical target for precision oncology strategies in EC.

The therapeutic relevance of epigenetic manipulation extends beyond endometrial cancer, as it has shown clinical benefits for multiple malignancies. In myelodysplastic syndrome (MDS) and acute myeloid leukemia (AML), DNMT inhibitors, such as azacitidine and decitabine, have demonstrated significant effectiveness by promoting cellular differentiation and restoring expression of silenced tumor suppressor genes [24,111,112,113]. Similarly, cutaneous T-cell lymphoma and other hematologic malignancies have revealed therapeutic effectiveness with HDAC inhibitors, including vorinostat and romidepsin [114,115]. In solid tumors, epigenetic treatments have also been demonstrated to promote tumor immunogenicity, increase antigen presentation, and sensitize solid tumors to be more susceptible to immune checkpoint inhibition and conventional chemotherapy [93,110]. Emerging evidence further indicates that epigenetic modulation can reverse therapy-induced resistant phenotypes, improve anti-tumor immune responses, and remodel tumor microenvironment in other cancers such as non-small cell lung cancer, colorectal cancer, and ovarian cancer [66,93,116,117,118]. Collectively, all these findings support the wider applicability of epigenetic-targeted treatment as a strategy to overcome tumor heterogeneity and therapeutic resistance across the heterogeneity in a variety of cancer types.

## 7. Conclusions

Within the ESRF model, epigenetic dysregulation in EC represents a functional regulatory layer that actively governs therapeutic response and tumor evolution under treatment pressure. Recognizing epigenetic states as dynamic and reversible determinants of treatment sensitivity shifts the clinical paradigm from static molecular classification toward adaptive therapeutic control. Targeting epigenetic mechanisms provides a strategy to reprogram tumor behavior, reverse resistance, and restore pathway function across DNA repair, oncogenic signaling, and immune response networks. When integrated with biomarker-driven patient stratification and longitudinal monitoring, these approaches enable more precise and responsive treatment strategies.

The challenge moving forward is not the identification of epigenetic alterations, but their translation into clinically actionable frameworks that guide therapeutic decisions in real time. Therapeutic resistance in EC is not solely a consequence of fixed genomic alterations but a reversible epigenetic state. Importantly, this framework enables not only classification of tumor states but prediction of their reversibility under targeted intervention, providing a functional basis for adaptive therapy design. The ESRF model provides a mechanistic basis for identifying, monitoring, and reprogramming these states. Integrating this model into clinical practice enables a shift from static treatment selection toward adaptive, state-guided therapeutic control.

## Data Availability

No new data were created or analyzed in this study. Data sharing is not applicable to this article.

## Abbreviations

The following abbreviations are used in this manuscript:

AKT	Protein kinase B
CDK	Cyclin-dependent kinase
cfDNA	Cell-free DNA
ctDNA	Circulating tumor DNA
dCas9	Catalytically inactive CRISPR-associated protein 9
DDR	DNA damage response
DNMT	DNA methyltransferase
EC	Endometrial cancer
EMT	Epithelial–mesenchymal transition
ER	Estrogen receptor
HDAC	Histone deacetylase
HAT	Histone acetyltransferase
lncRNA	Long non-coding RNA
miRNA	MicroRNA
MMR	Mismatch repair
mTOR	Mechanistic target of rapamycin
MSI	Microsatellite instability
ncRNA	Non-coding RNA
PI3K	Phosphoinositide 3-kinase
PR	Progesterone receptor
SWI/SNF	SWItch/Sucrose Non-Fermentable chromatin remodeling complex
